# Supplementary figures and images for: Development of a minimal KASP marker panel for distinguishing genotypes in apple collections
Source: PLoS One. 2020 Nov 30;15(11):e0242940. doi: 10.1371/journal.pone.0242940 (PMC7703965; doi:10.1371/journal.pone.0242940)

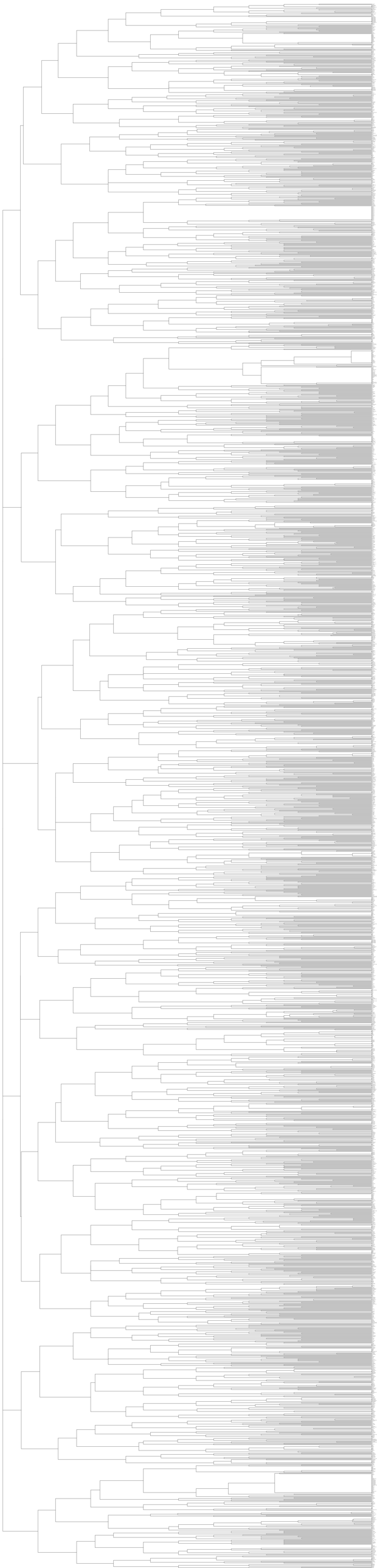

Supplement: S1 Fig — (PDF) [file pone.0242940.s002.pdf]
