# Supplementary material for: Development of a minimal KASP marker panel for distinguishing genotypes in apple collections
Source: PLoS One. 2020 Nov 30;15(11):e0242940. doi: 10.1371/journal.pone.0242940 (PMC7703965; doi:10.1371/journal.pone.0242940)
Supplement: Attachment — Submitted filename: Review_PONE-D-20-23044.pdf [file pone.0242940.s003.pdf]

Review of the Manuscript N°: PONE-D-20-23044

Manuscript Title: Development of a minimal KASP marker panel for characterising apple collections

Authors: Winfield M., Burridge A., Ordidge M., Harper H., Wilkinson P., Thorogood D., Copas L., Edwards K.J., Barker G.L.A.

This manuscript describes interesting results about the selection and validation of a set of 21 SNP markers for distinguishing genotypes in a large UK apple collection using the KASP technology. The article is well written and sufficiently detailed. The results seem robust. The discussion is sound even if some additional points could be further commented on. In the Experimental procedures, for each BAxxx KASP marker, the corresponding SNP_ID as defined by Bianco et al. (2016) for the Axiom®_Apple480K array from which the SNP have been selected through Harper et al's work (2019) should be detailed in order to allow complete tracing of the correspondence by the readers.

Additional comments and advices are detailed below.

General comments

- Discrimination: The described tool allow to distinguish the varieties tested here. But it would have been useful to really check the discrimination power of the tool for closely related individuals such as known full-sibs from controlled crosses (e.g., several full-sib progenies each represented by several individuals, especially when the parents are related thus generated inbreeding) or diploid parent-triploid offspring (especially when the parent has been inferred as the 2n gamete donor of the triploid offspring). Such full-sib relationships are quite frequent even in apple germplasm collections, or some triploid-diploid relationships have also been detected recently (see Muranty et al. 2020). This would have allow to assess the actual discrimination power (and the putative limits) of the proposed tool.

- Triploidy: nothing is said in the text about the possibility to distinguish the AAB and ABB clusters during the SNP calling process of triploid individuals. In Fig. 2, heterozygous triploids seem to be distinguishable from heterozygous diploids despite the clusters are quite close … In the present study, both genotypes are called AB as in the diploid case. It should be at least a little discussed with respect to Figure 2, especially considering the risk of misinterpretation of the various cluster boarders by the Kraken software. More important is the consequence (that should be expressed): as it is, the proposed SNP set does not allow to distinguish diploids from triploids. The heterozygosity rate discussed in the Discussion section is only a very preliminary proxy for that, whereas SSR markers are comparatively much more powerful. Heterozygosity rates ranged from 0.14 to 0.85 for diploids, and from 0.28 to 0.9 for triploids, which is actually very similar. It should be possible to compute the predictive power of diploidy versus triploidy accounting for the sole heterozygosity criteria: most probably, the predictive power would be very low, with many accessions incorrectly assigned. Assessing triploidy thanks to SNP markers have been shown to be feasible using the 20K array by Chagné et al. (2015; DOI 10.1007/s11295-015-0920-8) and this ref should be cited and commented, since the number of analyzed SNP is a key factor in assigning correctly ploidy level. This is a limit of the small set of SNP described here that has to be indicated/discussed.

- Null alleles: null alleles are quite frequent even in SNP markers and have already been invoked as a reason for Mendelian errors in pedigree (e.g. in Vanderzande et al. 2019 or in Muranty et al. 2020; see also Di Guardo et al. 2015, doi: 10.1093/bioinformatics/btv446). This hypothesis should be considered and discussed when considering the results obtained for markers BA07b and BA022 where more disagreement was observed between results obtained here and with SeqSNP platform. For BA07b, the 71 individuals with A:G with SeqSNP and C:C here could in fact be C:null (the corresponding "T" probe may have unknown additional SNP close to its 3' end for a significant part of the genotyped accessions, ad such additional SNP would not be

creating an issue with the SeqSNP approach). The same argument could be invoked for the marker BA022: the 181 individuals with C:T by SeqSNP would be C:null with the KASP approach. In both cases, C:null individuals should be located close to C:C individuals on the intensity graph, despite they could be located a bit lower on the X-axis (or Y-axis). To extend this interpretation, some of the miscalls observed for these 2 markers could actually be individuals with the genotype 'null:null' (and this could also be the case for other markers, albeit probably at a very low frequency).

- Error rate: the error rate has been computed by comparing the KASP genotyping data with the SeqSNP genotyping data of Harper et al. (2019). The comparison could be extended (for the common varieties) with the Axiom_Apple480K array genotyping data recently made available by Muranty et al. (2020) (*Availability of data and materiel:* https://data.inra.fr/dataset.xhtml?persistentId=doi:10.15454/IOPGYF).

Specific points:

Title:

The term 'characterising' in the title is probably excessive. Interestingly this term is only used in the title and not at all after in the text. The authors further use 'distinguishing', which term is much more adequate with regard to the actual capacity of the SNP set (e.g., 'characterizing' could be used if the SNP set would allow to perform robust genetic structure analysis or ploidy determination).

The title could be: 'Development of a minimal KASP marker panel for distinguishing different genotypes in apple collections'

Introduction

Line 25: ref 14, 19 and 22 could be added to the ref list: "(2, 4, 5, 14, 19, 22)".

Line 31: for apple, Bianco et al. 2014 (20K array: https://doi.org/10.1371/journal.pone.0110377) could be also cited. It is much cheaper than the 480K array and very efficient for distinguishing apple genotypes, checking the ploidy status (and even aneuploidy), and inferring relationships.

Line 41: add: "representing about 2,200 distinct … »

Line 41 and afterwards: better not to use the term 'lines'. "Line" is generally not used for apple germplasm description or genetic diversity … Much better to use 'accession' everywhere in the text and in the table/figure captions. In some situations, 'samples' or 'individuals' could be used instead of lines.

Line 44: clarify the acronym MUNQ for the first time use.

Results

Lines 59-60: the worksheet '*KASP probes*' of Supplementary Excel file S1 lacks some explanations and indications. Especially 'chromStart' and 'chromEnd' are a bit obscure and explanations are lacking for colored cells. Moreover, the original names (Affymetrix_id and SNP_id) of the 31 retained SNPs from the Axiom®_Apple480K array as specified in Bianco et al. (2016; https://onlinelibrary.wiley.com/doi/full/10.1111/tpj.13145) should be indicated in this worksheet. E.g., in the Table S1 (Supporting information) of Bianco et al. 2016, BA06 is corresponding to the following SNP:

Affymetrix_id	SNP_id	Customer_chr	Customer_pos
AX-115521188	FB_AFFY_9751091	MDC054245.000	30344

This correspondence (both IDs + Customer_chr + Customer_pos) must be indicated for each of the 31 SNP in order to allow the readers to follow the full pathway from the article of Bianco, through the cited article of Harper et al. 2019 to the present paper. In the Supporting information of Harper et al. 2019 (ppp310074-sup-0002-DatasetS1.xlsx), the concatenated Probe_ID (Customer_chr + Customer_pos) is indicated in worksheet 'Minimum Marker Set' and should be reported in the present paper together with the original Affymetrix and SNP IDs to explicitly describe the full pathway.

Line 68: Table 2 is too detailed to be maintained in the main text. All individuals with fails > 2 should be summed in order to get only 4 lines in the Table (0, 1, 2, >2 fails). The detailed version could be given in Supplementary information. Red color in Table 2 is thus no more useful.

Lines 80-81: Make a second sentence instead of a comment in brackets.

Line 97: what is "S1 Fig." ?? There is no figure in the pdf file: Supplementary_File_1.pdf. In the worksheet 'Ploidy and Heterozygosity' of the Excel file Supplementary_File_1.xls, there is no figure, but a screen mentions the following (??):

> This chart isn't available in your version of Excel.
>
> Editing this shape or saving this workbook into a different file format will permanently break the chart.

Line 100: ref "(12)" should not be mentioned here since it refers to a method that should be mentioned in the Experimental procedures section (lines 435-436).

Lines 103-104: the ref Harper et al. 2019 should be introduced earlier in the Introduction section.

Line 110: add '%'.

Lines 111-121: Null alleles? See general comment above.

Lines 122-123: The second part of this sentence is not a result but a comment and should thus be moved into the Discussion section. Only results in Result section of scientific articles …

Line 127: add "were expected to have ».

Lines 130-133: these two clusters should deserve a more accurate analysis to understand the reasons of the discrepancy and thus better specify the advantages and limits of either platforms. This aspect is fully in the scope of this article.

Line 142: correct '*Accessions Studied'* and '*Accessions with Nominal Reps*') instead of 'Lines studied" and "Accessions with Known Replicates" (in reference to the S1 table) (or worksheets '*Accessions with Known Replicates*' is missing in S1?).

Lines 146-168: Table 4: complete the writing of the caption: "The 'Odd One' column indicates …" ??
Also, the indicated numbers are not all correct: check everything again.
E.g., for the line 'N° Reps = 4': 'Identical'= 10 instead of 11, and 'Odd One' = 4 instead of 3
For 'N° Reps = 5': '2 SNPs diff'= 0 instead of 1
'N° Reps = 6' should not appear since it is not specified in the worksheet 'Accessions with Nominal Reps' of S1.
The issue with this table 4 is that some of the accession groups described in the worksheet 'Accessions with Nominal Reps' can be counted twice (or more) according to some counting rules which could be better described in the text … For example, for 'N° Reps = 4', the group 'Pren Glass' has to be counted in Odd One, but then could be counted also in the column '1 SNP diff' after having discarded 'Pren Glas3' of this group …

Line 164: 6 replicates is not present in worksheet ‘Accessions with Nominal Reps’: should be discarded from Table 4 …

In S1 worksheet ‘*Table 4*’ is equivalent to text Table 3: should be corrected.

Lines 186-187: this statement is a bit odd when considering the many synonyms found in apple trees. Perhaps it is the meaning of the term 'accession' that is again inappropriate here (?). The results described in the next sentence are fully expected in apple germplasm collections …

Lines 192-193: worksheet ‘*Paired Singletons*’ not found in S1? The section lines190-210 cannot thus be checked …
But filtering with MUNQ 163 in worksheet ‘*Accessions studied*’ indicated 21 accessions with this MUNQ (instead of 15 stated at line 197), and all but one (2032 – Vegi Cox) exhibit the same SNP profile when not considering the miscalls.
Also, filtering with MUNQ 901 indicated 19 accessions with this MUNQ (instead of 17 stated at line 199) and all exhibit the same SNP profile when not considering the miscalls.

Lines 204-208: even if these cases are rare, they should be considered more carefully since they could correspond to a limitation of the detection capability of the SNP set. A solution could be to run SSR markers to check the genotype with another type of genetic markers and to verify consistency with MUNQ assignment.

Lines 211-212: again, this sentence is not a result; it contains references and thus should be in the Introduction or in the Discussion sections. At least discard the references or introduce this paragraph differently.

Line 213: specify: “… reported to be tetraploid according to SSR markers (14).”

Lines 215 and 225: correct « S1 File: worksheet ‘Ploidy and Heterozygosity » (in accordance with S1 table)

Line 220: check sentence

Discussion

Line 235 (and elsewhere in the text): should it be called ‘error rate’ or ‘disagreement rate’. With error rate, the SeqSNP platform is seen as the truth, and the KASP as the fault maker … is it correct?! And the argumentation is more balanced at line 238 …

Line 264-266: this assertion is not supported by previously reported results …

Lines 271-272: similarly to a statistical test where you accept a false discovery rate (e.g., risk alpha = 5%), you could compute and indicate the risk probability of declaring two accessions distinct whereas they are actually not while exhibiting ‘n’ discordant SNPs as follows: (error rate)^n.
According to the error rate you consider (line 259: 0.42% - 2.9%), this probability will vary.
E.g., for a conservative assessment (error rate = 2.9%), the risk probability of false discrimination between 2 accessions with 2 discordant SNPs is: (0.029)^2 = 0,000841
This computation does not account for close relatedness … Other more elaborated statistics exist.

Lines 297-298: it could also be the reverse situation, i.e. an error in SSR data or in collecting the correct material at the time of leaf collection by F. Fernandez-Fernandez …! Here again, where is the truth? : in SSR data or in SNP data ? It can vary from case to case …

Line 302: first, these results should be previously indicated in Results section and not only here; second, this worksheet ‘*DEFRA comparison*’ is not present in Excel table S1 …

Lines 304-309: again this paragraph should be in the Results section and not here as it is. Then, what is your interpretation (Discussion section) of these results??

Lines 327-330: the preferential position of many triploids in some particular clusters is probably a matter of chance. Moreover the clustering of triploids and their position in the dendrogram rely on "incomplete" genotyping data (since the AAB and ABB genotypes are gathered in a single genotypic class AB). Their position should therefore be considered with caution.

Line 342: SNP-array-based genotyping is indeed more expensive than the simple set of 21 SNPs described here but is however much more informative. For example, pedigree relationships can be inferred from the 20K array, which will not be the case with only 21 SNP.

Lines 345-347: the indicated costs most probably only contain the technical costs and not the salary costs. It should be stated, with some indication of the time necessary if possible.

Experimental procedures

Again, better to use 'accessions' or 'samples' instead of 'lines'.

Line 360: correct "Accessions Studied" (not 'Lines')

Lines 361-362: if different trees with the same variety name were collected "from different orchards or gardens", they should be called different accessions …

Lines 376-377: some minimal reference to the paper of Bianco et al. (2016) from where the polymorphic and robust SNP markers were initially extracted for the work of Harper et al. (2019) could be included here.

Line 387: 'each of the 21 markers' is not previously detailed. Why 21 ? At least, indicated 'For each of the 21 selected markers' to specify they directly derive from the previous step.

Lines 387-390: as stated before, the accurate correspondence between the 21 selected SNP markers and the SNP_ID initially established in Bianco et al. (2016) should be clearly indicated beside the new names used here. E.g., BA06 (SNP_id = FB_AFFY_9751091 = AX-115521188).

Line 389: in S1, only 'KASP probes' is indicated for the worksheet: harmonize.

Comments on Supplementary File 1 (Excel table S1):

In S1, accession numbers seem not to coincide with the NFC coding system, i.e. year-number: e.g. 2000-008 or 1966-148 for Cox's Orange Pippin (LA): http://www.nationalfruitcollection.org.uk/full2.php?id=1330&&fruit=apple). Exact accession numbers (Accessions ID) should be added as an extra-column in S1 worksheets '*Accessions studied*' and '*Accessions with > 2 SNPs failed*' (and '*Ploidy and Heterozygosity*') for allowing other research teams to further elaborate on the published SNP dataset.

Worksheet '*Accession studied*': what is the meaning/added value of the Notes from Matt Ordidge and Mike Winfield?
Lines 14 and 15 of this worksheet seem inverted between Original names and Notes (M. Ordidge): i.e., see Adams Pearmain / Adamsapfel Ost.
Same for lines 33-36 (Alkmene ➔ Alliston Pippin): switches with Notes (M. Ordidge)
Probably useful to check the whole worksheet …

Worksheets '*Accession studied*' and '*Ploidy and Heterozygosity*' seem to contain the same genotypic information for the 21 SNPs analyzed: useful to duplicate the information?
If the authors maintain the genotypic data only in the worksheet '*Ploidy and Heterozygosity*', the accession code (Accession ID) should be duplicated in that worksheet to avoid misinterpretation with the column 'Name' (slight spelling differences, etc).

Supporting information

Line 549: the furnished S1 Fig does not give any dendrogram but the copy-paste of an Excel file (close to the content of worksheet 'Accessions studied') … ? ➔ Error when uploading this file?
