# Supplementary material for: Development of a minimal KASP marker panel for distinguishing genotypes in apple collections
Source: PLoS One. 2020 Nov 30;15(11):e0242940. doi: 10.1371/journal.pone.0242940 (PMC7703965; doi:10.1371/journal.pone.0242940)
Supplement: Attachment — Submitted filename: Re-review#2.pdf [file pone.0242940.s005.pdf]

2d review of the Manuscript N°: PONE-D-20-23044_R1

New manuscript Title: Development of a minimal KASP marker panel for distinguishing genotypes in apple collections

Authors: Winfield M., Burridge A., Ordidge M., Harper H., Wilkinson P., Thorogood D., Copas L., Edwards K.J., Barker G.L.A.

General comment: the comments previously sent in the 1st review have been considered, integrated or commented.

Minor, but important points still to be considered are:

Line 117 : « here had been genotype previously" : correct 'genotyped'

Replicates: looking at the comments of Reviewer#1, I suspect there is an issue with the term 'replicate' which is used in different ways according to the situations and sentences.

I propose that the authors use 'technical replicates' for sample replicates from a same tree, such as for the cultivar Yeovil Sour (Lines 66-67), and 'biological replicates' when samples come from different trees (mostly with the same names). Then, the authors should clearly define in Mat&Meth what they call 'nominal replicates', which adjective suddenly appears in lines 240 and 310 for sheets of Supplementary File S1 and in the caption of Table3 without having being previously defined … This would help clarifying the terms used along the manuscript.

Lines 122-123, I would propose to change accordingly: "True replicate samples were expected to have 21 identical SNP calls; any SNP difference between technical or biological replicates was assumed, therefore, to be the result of an error in calling …"
Line 131: "same name could be taken as putative biological replicates …"
Line 134: "that had fewer than 3 SNP differences could be taken as genuine biological replicates."
Lines 148-149: "the two samples of Kim are not true biological replicates …"
Line 239: " … singletons and 204 with nominal replicates …"
Lines 244-246: "the number of samples with biological replicates … when samples have known nominal replicates, e.g. the sample Black Vallis 1 »
Line 304: "The samples with true technical or nominal replicates allowed us .." (since the 1st example is for Yeovil Sour where technical replicates have been sampled)
Lines 308-336: again it is biological or nominal replicates and not technical ones …
Line 311: "the potential nominal replicates were collected from different trees, often from different orchards or gardens. » : it fits exactly with the definition of a biological replicates : different samples from different tress, and not from a same tree.
Lines 312-314: "The ten nominal replicates of Cox's Orange Pippin were identical to each other, as were the eight nominal replicates of Breakwell's Seedling, and the seven of Frederick. However, some nominal replicate samples did not have identical SNP profiles …"

Lines 317-318: "In 11 cases (8.8%), potential biological (nominal) replicates had one SNP difference and in two cases (1.6%) the potential biological replicates had two SNP differences …"
Including the adjective 'potential' (or 'deemed'?) has the advantage to introduce the idea that the samples are expected to be biological (nominal) replicates, but it's not always the case … :
Line 324: "nine of the potential biological replicates (i.e., by name = 'nominal') from the NFC were confirmed not to be so" (instead of the term 'apparent' …)
Line 344: why using 'named replicates' here instead of 'nominal replicates' ??
Line 355: "In total, we had 563 accessions with true nominal replicates ~~by name~~, …"
And so on …

Line 105-106: "primer sequences, together with their corresponding SNP Affymetrix code from Bianco et al. (2016) are available in File S1: spreadsheet '*KASP probes*'. It's very important to clearly state and highlight here the correspondence between the new KASP probes and the previous Affymetrix SNP codes

Lines 137-138: since additional MUNQ codes have been provided through the new cited reference n° 12, this reference should be used here instead of 'personal communication': "additional MUNQ codes, created as part of an extension of the worked initiated in (11, 16), were provided by Caroline Denancé, INRAE, Angers, France (12)."
Similarly Line 325: "… been genotyped previously (20, 21) and had been assigned unique codes, MUNQ codes (11, 12) …"

Lines 148-149: "the two samples of Kim are not true biological replicates and we only have two true nominal samples of Gavin"

Lines 184-185: "The sequences of these 21 KASP probes, along with the corresponding SNP Affymetrix code (AX……) from Bianco et al. (2016) and the chromosomes to which they are assigned, are given in File S1" (same argument as before to allow the reader to trace the correspondence from the paper of Bianco et al. (2016) through the paper of Harper et al. to the current one …)

Line 409: "according to analysis with flow cytometry and/or SRS markers (20)" : I guess the authors mean SSR.

Lines 423-431: simplify caption of Fig. 5 :
- The following sentence "In most cases, triploid accessions correspond with high heterozygosity; there is a tendency for these samples to be found in clusters 4 (light blue) and 7 (red)" should be discarded and included in the Result section: a figure caption does not have to contain any result interpretation … !
- Same issue for the two following sentences: "**B)** There was a statistically significant difference between the mean heterozygosity of triploids and that of the other three categories as determined by one-way ANOVA ($F(3,2478) = 206.3$, $p = 2.2e\text{-}16$). The unknown samples ranged in heterozygosity from very low to very high." All that text corresponds to result interpretation, not to a fig. caption describing the figures … !!

Lines 446-455: discussion about the possible null allele issue should be at least evoked here for BA07b, BA22 (and BA08) ! The term 'null allele' is yet never mentioned in the text whereas it's a commonly used term for SNP issue in apple genetics.

Lines 466-468: "Also, additional polymorphisms surrounding the target SNP may reduce probe hybridization efficiency resulting in decreased signal intensity from the fluorochrome, and possibly to null allele (11,25)."

Lines 471-474 ("shared missing marker BA08 in the accessions with MUNQ code 901 and the shared missing marker BA06 in the accessions with MUNQ code 803") looks to be a summarized description of newly-added paragraph in Lines 481-486 : please, simplify.

Line 529: "… were able to detect a difference in heterozygosity between diploids and triploids…" (and not tetraploids, I guess ?!).
