# Supplementary material for: Development of a minimal KASP marker panel for distinguishing genotypes in apple collections
Source: PLoS One. 2020 Nov 30;15(11):e0242940. doi: 10.1371/journal.pone.0242940 (PMC7703965; doi:10.1371/journal.pone.0242940)
Supplement: Attachment — Submitted filename: Response_to_Reviewers.docx [file pone.0242940.s004.docx]

**Reviewer 1**

*LINE 104 -(Harper et al., 2019)., reference number?
LINE 293: Fernandez-Fernandez, 2010, reference number?
Line 296: Fernandez-Fernandez, 2010, reference number?
LINE 323: Larsen et al (2018), reference number?*
Corrections have been made to these citations

*LINE 354: 2,675 trees representing more than 2,200 distinct accesions: 2,104 (National Fruit Collection at Brogdale in Kent) + 153 (National Botanic Garden of Wales) + 504 various orchards and gardens = 2104+153+504 = 2,761 trees?.*

*LINE 42: 2,143 lines from the National Fruit Collection (NFC) , 2,143 or 2,104?*

*LINE 360: “For 229 varieties, samples were taken from more than one listed under the variety name (often these 362 were from different orchards or gardens)”. In Sup. File 1 I see: 125*2 + 47*3 + 16*4 + 12*5 + 1*7 + 1*8 + 1*10 + 1*23 = 204 varieties. I suppose that the rest of the varieties have failed, but it is not clear, a basic table is necessary where the collected material is seen very clearly, the number of accessions of each collected variety, and the material that has failed, with final sums where You can quickly and clearly see the different numbers of samples that are discussed throughout the paper. To be understandable, everything must be detailed in a single table, because otherwise it makes understanding very difficult*

Thank you very much for pointing out our elementary errors in arithmetic. We had mixed up figures based on all samples sent to us and those that had at least 19 successful SNP calls. After the first mention of the 193 samples that were removed, in the ‘*Genotyping accuracy*’ section of the manuscript and Tables 1 and 2, we no longer include them in the numbers reported. We believe that this has removed the confusion.

*LINE 377: “markers capable of differentiating all 380 cultivars used in that study”. The 380 cultivars are those studied by Harper (2019)? because line 359 talks about 372.*

Again, thank you for this. The numbers has been corrected to 372.

*LINE 404:Estimating error rate and identification of mislabelled samples:
very important aspect . It is very difficult to understand the permissible error. It is an aspect that I do not understand at all, especially in species such as the apple tree, where the original mutations are very frequent, and that has given rise to numerous varieties that present very few, but visible, differences with the originals.
When studies with SSRS, a difference between two accessions in a single microsatellite is already enough to consider them as different accessions or varieties
I understand that the samples with repetitions or at least some of them, have also been studied with microsatellites (line 417: “Most of the accessions obtained from
Brogdale and the Welsh Collection have been genotyped previously using microsatellite or DArT markers or both”). Please show the comparison between microsatellites and SNPs. Those samples that show variation in Snps, did they show an identical profile using microsatellites or did they already show some variation?
This is a very important and fundamental aspect, especially when identifying close varieties, due to the high mutation rates present in apple trees, with SSRS a difference in a single marker already leads to consider that they are two different varieties.*

We are not saying that there is ‘permissible error’ but are simply recognising the fact that all techniques have an error rate. Thus, if two samples we believed to be identical differed by a single SNP we would want to repeat the assay to eliminate the possibility of this resulting from an error in scoring. No one would make an important decision on the basis of a single result!

Remember, apple varieties are clonally propagated by grafting.

Most of the accessions studied (1954) had a MUNQ code. The whole purpose of this manuscript is to show that samples that were identical using SSRs (Fernandez-Fernandez, 2010) also proved to be identical using our SNP panel. However, this only becomes completey apparent when MUNQ codes are used rather than names (the three samples named ‘*Strawberry Pippin*’ highlight this point; they had three very different SNP profiles and, in the end, proved to be three different varieties (MUNQ codes 2072, 2430, 2532). The comparison between our results and SSR is based on the samples with MUNQ codes; these accessions have been analysed with SSR markers

At no point in the manuscript do we suggest that accessions with different SNP profiles are the same variety. We do show the converse, however; accessions with the same name may prove to be quite different from each other (they are merely homonyms). Also, accessions that have quite different names might prove to have identical SNP profiles (that is, they are synonyms).

*Of the 204 accessions that present repeats, how many have coincided for the total of 21 SNPs?

Please explain this aspect very clearly, and compare the data with the results obtained through SSRs.*

We have included an extra spreadsheet in File S1 (‘*Nominal Singletons with Reps*’) that shows the correspondence between our results and those obtained with SSRs.

*LINE 83: “One can adjust for these experimental errors when samples have known replicates, e.g. the sample Black Vallis 1 differed by one SNP (BA14) from the other two Black Vallis samples creating two distinct genotypes where we presume there should be only one”
the same problem as before, why is it known that they are the same? showed the same profile by SSRs? If so, how reliable are the SNPs considering two accessions that differ by a marker, when they are equal by SSRs, and also knowing that there are varieties that differ only by one allele, and that the species mtation rate is elevated? Is it a system that can be used with total reliability with that margin of error that is not found with SSRs? Is hard to understand*

We agree with you entirely here. What we are saying is that there appears to be an error is scoring; in a real world situation one would repeat the genotyping of these three samples. If the ‘error’ persisted then it would no longer be considered an error but a genuine difference between the samples.

*LINES 113-123 “we expected complete concordance between the SNP profiles produced by the two assays”…..” the overall concordance between the two platforms for all 21 markers was 95.8%”
I am understanding from this explanation that the technique is reproducible in a percentage of 95.8. Is correct? How would the case of a litigation on a plant patent with a very small genetic difference be resolved if the reproducibility is not 100%?*

The error rate you quote is related to the concordance between the scores given with our minimal SNP markers set (assayed using KASP) and those produced by a SeqSNP approach (a sequencing based approach where SNPs are called automatically). Our concordance between the two approaches is as good as, if not better, than that of the team that developed SeqSNP. This is stated in the manuscript and a citation given.

As for litigation for a plant patent: we have not developed this tool for such a situation but for use in maintaining and renewing orchards as is stated in the manuscript’s Introduction.

*LINES 135- 168: Accuracy of scoring replicates.
It is not well understood, the replicate samples, had they been tested by SSRs, and by SSRs they were the same and now they do not match? and those that do not match are removed from the analysis? or using SSRs they did not match and there were differences between them?*

The replicate samples are just that; leaves samples were collected from several trees that had the same name. We carried out our analysis completely blind to the sample names and MUNQ codes. Thus, when we found samples that shared the same SNP profile we checked to see whether they had the same name (at which point we calculated an error rate - but we know that this is too high because of mislabelling of samples and homonyms) and then the same MUNQ codes (we calculated the error rate a second time to take into account results from SSR and DArT markers - these form the basis of the MUNQ codes). In doing so we discovered that our SNP results correlated almost perfectly with the MUNQ codes but not with the names given to them. That is our results were able to distinguish varieties that SSRs and DArT markers were able to distinguish and produced identical profiles for accessions that SSRs indicated were identical. Please note, we didn’t carry out any SSR or DArT analysis, but are comparing our results with the DEFRA report of 2010 (Frenandez-Frenandez) and the MUNQ codes provided by authors on the Muranty 2020 paper.

(*Malus* Unique Codes: unique codes assigned to apple varieties; full MUNQ listing are available at https:doi.org/10.15454/HKGMAS). This has now been cited in the manuscript.

All the samples that have been given a MUNQ code have been scored using SSRs and, in some cases but not all, DArT markers. Indeed, this is the basis for them being assigned a unique code. Thus, when we consider our results with respect to the MUNQ codes we expect to see identical SNP profiles for those accession that have the same MUNQ code regardless of the names that were supplied with the samples at the time of collection. This is, indeed, what we do see.

*LINE 145-150. How is it possible that two identical samples do not show the same profile and what reliability can be given to the analysis when entering a new sample to identify it? because there will be more unrelated samples that differ only by two Snps, and are not considered equal, when will we consider them equal if we do not have the names?
Stated in another way, if it was not known that they were replicated a priori, they would be considered synonymous samples showing two different Snps if the previous information was not had??*

We are saying that two identical samples should have identical SNP profiles. In the rare cases when this doesn’t appear to be the case we suggest that the samples are either i) not identical due to mislabelling (this could be in the orchard or at any point after collection of leaf samples and subsequent genotyping, or ii) that an error in calling the genotype has occurred (error rate between 0.18 - 2.5%). In this article we are simply establishing the efficacy and reliability of the minimal marker approach and showing that it is comparable to that of SSRs but cheaper.

The error rate you quote is related to the concordance between the minimal SNP markers (assayed using KASP) and an automated sequencing based on SeqSNP.

If we given two unknown samples and wanted to be certain that they were identical, I would run the samples perhaps three times each in order to eliminate the possibility of a scoring error leading to the wrong conclusions.

*LINES 193-196: “At first sight, this might appear to indicate that our primer panel is not able to discriminate varieties. However, when the groupings were considered in the knowledge that a number of genotypes were replicated in the form of clones and sports, most of these issues could be resolved.”
What does the method contribute if one does not start from previous information on the accessions? If we start from 1000 accessions for which we have no previous information, how sure are we that we make a correct identification and that all synonyms or mutations are identified?*

We agree entirely with your statement; one normally considers available information prior to conducting an experiment. However, because we were developing a new approach, we wanted to genotype ‘blind’ to the sample names so that it didn’t influence in any way our SNP calling. Thus, only when we aligned sample names, MUNQ codes and SNP profiles were we in a position to determine whether our set of 21 markers had given results comparable with those produced with SSRs. In writing the manuscript we wanted to follow the steps of producing clusters on the basis of SNP profiles and only then seeing how this compared to sample names and then MUNQ codes; at each new reveal anomalies and inconsistencies reduced.

LINES 206-208: “In those cases where differently named accessions that had identical SNP profiles had been collected entirely from small orchards or private gardens, no conclusions about the groupings could be drawn.”
Are they not considered synonymous if they present the same SNPs or by microsatellites were they different? or the information with 21 SNPs is not enough to establish that they are true synonyms?

We agree with you, the sentence is rather clumsy and vague (we have now changed this). What we are saying here is that if we have two samples each with a different name but with the same SNP profile, in the absence of MUNQ codes, which samples from small local orchards do not have, we cannot determine which of the two names, if either, is the correct one. However, if a sample from a small, local orchard shares a SNP profile with a sample that does have a MUNQ code we can provisionally assign it a name. Obviously, before definitely changing the name of an accession it would be important to repeat the assay (although our SNP calling has a very low error rate - see our many articles on genotyping in wheat).

*LINES 270-272: “If, as we maintain, error rates are low, any two samples that differ in their genotypes by more than one or two SNPs must be considered genuinely different”
With SSRs with a difference in a micro, two varieties are already considered, why not with SNPs?. If we have two unknown samples, or named differently, and they differ by a single SNP, are they also considered genetically identical even though they were not collected with the same denomination?*

We agree entirely, if two samples differ by a single SNP they should be considered as two different varieties. However, before coming to that conclusion one must be sure that the difference is genuine and not the result of an error in calling. We would use the same logic for any marker system SNP, SSRs, RAPDs, AFLPs

*LINE 210.* *Heterozygosity and ploidy Why is ploidy not known out of 92 samples?*

This is simply a matter of fact. Not all apple accessions have been assessed for ploidy; we report the ploidy only in cases where we have records to support this.

*LINE 242-245: “Third, samples that were believed to be replicates (that is, they either shared the same name or, where available, the same MUNQ code) were tallied to determine how often they had identical genotypes; with a small degree of error, replicate samples had identical genotypes”

Is this error in the identification of collections permissible, and especially in the event that a legal conflict arises about different similar varieties, and that they only differ due to a somatic mutation, or is this method not recommended for that purpose?*

As stated above, this method is not intended for the resolution of legal cases. It certainy could not identify somatic mutation, but nor could a limited panel of SSRs. One would have to use a tool, such as sequencing, that gives coverage of the whole genome (c. 750 Mb). And, don’t forget, that in a long lived plant species such as a tree, it is highly probable that two leaves taken from different parts of the canopy my well differ because of somatic mutation (our group reported on this in Black Poplar in 1997).

Our SNP panel is designed for genotyping germplasm collections.

*LINES 270-272: “If, as we maintain, error rates are low, any two samples that differ in their genotypes by more than one or two SNPs must be considered genuinely different”
With SSRs with a difference in a micro, two varieties are already considered, why not with SNPs?. If we have two unknown samples, or named differently, and they differ by a single SNP, are they also considered genetically identical even though they were not collected with the same denomination?.*

Again, we are presenting a new ‘tool’ for genotyping germplasm collections (in this case apple, but this approach is being used by our group for other species; e.g., Wheat and Barley) and, given that all approaches have an error rate, we are trying to establish our confidence intervals. Thus, if we were to consider two samples that had one SNP difference on the basis of one assay, in the absence of any other information, we couldn’t with 100% confidence say they are genuinely different. However, if samples had 3 or more differences we would be highly confident that they are different because our error rate is not that high.

*LINE 387-388: “For each of the 21 markers, two allele-specific forward primers and one common reverse 388 primer were designed using the same primer sequence used in Harper et al. (2019). (S1 File: 389 ‘KASP probes as used’).”. looking at the table, it is seen that there are 31, I understand that the 10 that do not have a name are those that Harper used in his work, and that they are not used in the present work, but it is necessary to clarify it in some way, it does not make sense to see some markers, without a name or anything, you don't know what it is*

We’ve reordered the columns in the Excel spread sheet so that it is clear that all primer combinations have a name (e.g., KASP Apple 1). We have also added the Axiom ID codes (allows one to trail back to Bianco *et al.*, (2016) and the SeqSNP codes (allows one to trail back to Harper *et al.,* (2019)).

Supplementary Data: we did have problems loading some of the files and it would appear that you have been viewing a file that has some columns that should have been removed.

*2- Do the authors think with certainty that from the merely agronomic and identification point of view, without taking into account the economic cost, SNPs provide more solid and robust information than SSRs?
Can it be categorically stated that only using 21 SNPs allows correct identification of all varieties be made, detecting somatic mutations? Is it possible to correctly identify and classify the varieties without having any prior information about them?*

We believe that our SNP panel is as reliable as SSR markers in identifying accessions. And, indeed, that is exactly what this manuscript is showing; all accessions with a MUNQ code (based on earlier studies with SSRs and DArT makers) had distinct SNP profiles; conversely, where we had been given identically named sample with different MUNQ codes (the three samples named Strawberry Pippin), we identified these as being distinct. Thus, if you were to present us leaves from all c. 2,000 samples from the Brogdale collection, we would be able to tell you the trees from which they were taken. Our results would be just a reliable as those that would be produced using SSRs.

What is more, neither SSRs or SNPs assay the whole genome so neither approach is able to identify small differences between accessions. The article by Fernadez-Fernandez (cited in the manuscript) clearly point out that colour variants, for example, are often identical when genotyped with SSRs; unsurprisingly, they also had the same SNP profile.

*How would the case of a dispute over a plant patent with a very small genetic difference be resolved? would it be possible with 21 SNPs?*

We do not propose using our minimal marker approach in such a cases. They have been designed to help in the management of germplasm collections, as we say in the Abstract and Introduction (‘*Accurate identification of named accessions in germplasm collections is extremely important, especially for vegetatively propagated crops’*). However, having said that, in a legal case, if one really wanted to establish that two samples were the same I think it would be better to sequence rather than genotype with a small number of markers be they SSRs or SNPs. No marker system can tell you whether there is or isn’t a difference in sequence that hasn’t been assayed. Indeed, somatic mutations would almost certainly mean that, in a long lived tree species, if one were to take leaves from two distant parts of the canopy, small sequence differences might well be seen. As a group, using AFLPs to study black poplar (*Populus nigra*), we reported on this in 1997.

**Reviewer 2**

*- Discrimination*

These is a very good suggestion and, as we further develop our minimal marker approach, we will keep these ideas in mind. Our scope in this study was to show that the software tool (available on github) was able to generate a valid, minimal marker set and practically show that it works. As a tool for distinguishing apple genotypes, I think it does. If we were to be studying inbred lines, we might want to expand the marker set.

*- Triploidy*

Thank you very much for this suggestion. We are certainly well aware of the problems of scoring SNPs in polyploid species as we do most of our work with bread wheat. We have added a sentence to the Discussion pointing out that there may well be a difference between the clusters formed by AAB individuals and ABB individuals.

Although we had pointed out the limitations or using our small SNP panel to make conclusions about heterozygosity, we have added an additional sentence to the Discussion stress this limitation. We have included the citation Chagné et al. 2015, and we are happy to do so, because it draws attention for the need for a larger number of SNPs when one is attempting to draw conclusions about biology. We are presenting the

*- Error rate*

Indirectly our study is making a comparison with the Muranty *et al.* (2020) results: the MUNQ codes were provided to us directly from authors on that paper. Much of the ‘error’ we report is related to the names given to the accessions. We wished to carry out the study blind to the MUNQ codes and this we did. After genotyping we created clusters of identical SNP profiles and then projected the names onto them. This represented the first error we report (it was disturbingly high for our liking!). Only then did we turn to the MUNQ codes and find that almost all errors (naming, sampling, technical, whatever) we removed. We wished to capture this approach within the manuscript.

Specific points:

*Title: The term ‘characterising’ in the title is probably excessive. Interestingly this term is only used in the title and not at all after in the text. The authors further use ‘distinguishing’, which term is much more adequate with regard to the actual capacity of the SNP set (e.g., ‘characterizing’ could be used if the SNP set would allow to perform robust genetic structure analysis or ploidy determination). The title could be: ‘Development of a minimal KASP marker panel for distinguishing different genotypes in apple collections’*

We agree entirely with your reasoning and suggested alternative; the title has been changed.

*Line 31: for apple, Bianco et al. 2014 (20K array: https://doi.org/10.1371/journal.pone.0110377) could be also cited. It is much cheaper than the 480K array and very efficient for distinguishing apple genotypes, checking the ploidy status (and even aneuploidy), and inferring relationships.*

Citation added.

*Line 41: add: “representing about 2,200 distinct ... » Line 41 and afterwards: better not to use the term ‘lines’. “Line” is generally not used for apple germplasm description or genetic diversity ... Much better to use ‘accession’ everywhere in the text and in the table/figure captions. In some situations, ‘samples’ or ‘individuals’ could be used instead of lines.*

Change made as requested.

*Line 44: clarify the acronym MUNQ for the first time use.*

A definition of the term has been inserted.

*Results Lines 59-60: the worksheet ‘KASP probes’ of Supplementary Excel file S1 lacks some explanations and indications. Especially ‘chromStart’ and ‘chromEnd’ are a bit obscure and explanations are lacking for colored cells. Moreover, the original names (Affymetrix_id and SNP_id) of the 31 retained SNPs from the Axiom®_Apple480K array as specified in Bianco et al. (2016; https://onlinelibrary.wiley.com/doi/full/10.1111/tpj.13145) should be indicated in this worksheet. E.g., in the Table S1 (Supporting information) of Bianco et al. 2016, BA06 is corresponding to the following SNP: Affymetrix_id SNP_id Customer_chr Customer_pos AX-115521188 FB_AFFY_9751091 MDC054245.000 30344*

The codes from the Bianco paper have been added, also the codes for the SeqSNP study. The terms ‘chromStart’ and ‘chromEnd’ have been removed and replaced with SNP position. We have also removed the primers that were not used in the final study (we had sent out a working file for Supplementary data and these are not included at all in the study).

*This correspondence (both IDs + Customer_chr + Customer_pos) must be indicated for each of the 31 SNP in order to allow the readers to follow the full pathway from the article of Bianco, through the cited article of Harper et al. 2019 to the present paper. In the Supporting information of Harper et al. 2019 (ppp310074-sup-0002-DatasetS1.xlsx), the concatenated Probe_ID (Customer_chr + Customer_pos) is indicated in worksheet ‘Minimum Marker Set’ and should be reported in the present paper together with the original Affymetrix and SNP IDs to explicitly describe the full pathway.*

Codes have been added.

*Line 68: Table 2 is too detailed to be maintained in the main text. All individuals with fails > 2 should be summed in order to get only 4 lines in the Table (0, 1, 2, >2 fails). The detailed version could be given in Supplementary information. Red color in Table 2 is thus no more useful.*

The table has been modified as requested.

*Lines 80-81: Make a second sentence instead of a comment in brackets.*

Altered as suggested.

*Line 97: what is “S1 Fig.” ?? There is no figure in the pdf file: Supplementary_File_1.pdf. In the worksheet ‘Ploidy and Heterozygosity’ of the Excel file Supplementary_File_1.xls, there is no figure, but a screen mentions the following (??):*

Figures have been uploaded correctly this time, we believe.

*Line 100: ref “(12)” should not be mentioned here since it refers to a method that should be mentioned in the Experimental procedures section (lines 435-436).*

Citation removed.

*Line 110: add ‘%’.*

Added.

*Lines 122-123: The second part of this sentence is not a result but a comment and should thus be moved into the Discussion section. Only results in Result section of scientific articles ...*

Sentence has been truncated as suggested.

*Line 127: add “were expected to have ».*

Added ‘to’

*Line 142: correct ‘Accessions Studied’ and ‘Accessions with Nominal Reps’) instead of ‘Lines studied” and “Accessions with Known Replicates” (in reference to the S1 table) (or worksheets ‘Accessions with Known Replicates’ is missing in S1?).*

Correction made.

*Lines 146-168: Table 4: complete the writing of the caption: “The ‘Odd One’ column indicates ...” ?? Also, the indicated numbers are not all correct: check everything again. E.g., for the line ‘N° Reps = 4’: ‘Identical’= 10 instead of 11, and ‘Odd One’ = 4 instead of 3 For ‘N° Reps = 5’: ‘2 SNPs diff’= 0 instead of 1 ‘N° Reps = 6’ should not appear since it is not specified in the worksheet ‘Accessions with Nominal Reps’ of S1. The issue with this table 4 is that some of the accession groups described in the worksheet ‘Accessions with Nominal Reps’ can be counted twice (or more) according to some counting rules which could be better described in the text ... For example, for ‘N° Reps = 4’, the group ‘Pren Glass’ has to be counted in Odd One, but then could be counted also in the column ’1 SNP diff’ after having discarded ‘Pren Glas3’ of this group ... This chart isn't available in your version of Excel.Editing this shape or saving this workbook into a different file format will permanently break the chart.*

We have completed the broken sentence and made corrections to the table.

*Lines 186-187: this statement is a bit odd when considering the many synonyms found in apple trees. Perhaps it is the meaning of the term 'accession' that is again inappropriate here (?). The results described in the next sentence are fully expected in apple germplasm collections ...*

For those potential readers of the manuscript not familiar with the naming of apples, we were trying to emphasise the fact that the names are a very poor indication of relationship.

*Lines 192-193: worksheet ‘Paired Singletons’ not found in S1? The section lines190-210 cannot thus be checked ... But filtering with MUNQ 163 in worksheet ‘Accessions studied’ indicated 21 accessions with this MUNQ (instead of 15 stated at line 197), and all but one (2032 – Vegi Cox) exhibit the same SNP profile when not considering the miscalls. Also, filtering with MUNQ 901 indicated 19 accessions with this MUNQ (instead of 17 stated at line 199) and all exhibit the same SNP profile when not considering the miscalls. Lines 204-208: even if these cases are rare, they should be considered more carefully since they could correspond to a limitation of the detection capability of the SNP set. A solution could be to run SSR markers to check the genotype with another type of genetic markers and to verify consistency with MUNQ assignment.*

All the lines from Brogdale with MUNQ codes have been genotyped using SSRs and our results for these lines (MUNQ code 163 and 901) are in complete agreement. The discrepancy is related to the names used for the accessions, and this is the very point we are trying to make: names are a very poor indication of the accessions in hand. Indeed, we have 10 lines that have the name ‘Cox,s’ (7 are from Brogdale and have the same MUNQ code, whilst 3 are from other orchards and do not have a MUNQ code - all 10 have identical SNP codes). There are 11 other accessions collected as singletons (by name alone); these were collected from Brogdale and had the MUNQ code 163 so should be identical to the Cox’s. All, except ‘Vegi Cox, had the same SNP profile as the Cox’s. In addition, there were also 5 Queen Cox accessions (3 from Brogdale (MUNQ code 163), 2 from other orchards), so these are not nominal snigletons, which also had identical SNP profiles. Finally, there were 5 UNKNOWNs that also had identical SNP profiles to that of the Cox’s. In total, then, there were 31 accessions that had the same SNP profile, 21 of which had MUNQ codes. We would suggest that all of these are the same variety except for the accession of Vegi Cox. The latter has a SNP profile so different to that of the others that it is almost certainly and error of some sort - most probably a sample mix up.

Similarly, for the samples with MUNQ code 901. There were 17 accessions collected as nominal singletons and 2 that had the name ‘Jonagold’ (a total of 19, but, as we say in the manuscript, 17 nominal singletons). All 19 of these had identical SNP profiles.

The dendrograms are particularly helpful for visualising these relationships. We are sorry that you didn’t have access to the dendrogram - this we submitted as a pdf file because it can be searched. Unfortunately, PLOS ONE only accepts figures as .tif files and so didn’t make our file available.

We have modified this section of the manuscript to try and make the issue a little clearer.

*Line 213: specify: “... reported to be tetraploid according to SSR markers (14).”*

Modified the sentence to include reference to the techniques used to establish ploidy.

*Lines 215 and 225: correct « S1 File: worksheet ‘Ploidy and Heterozygosity » (in accordance with S1 table)*

Correction made.

*Discussion Line 235 (and elsewhere in the text): should it be called ‘error rate’ or ‘disagreement rate’. With error rate, the SeqSNP platform is seen as the truth, and the KASP as the fault maker ... is it correct?! And the argumentation is more balanced at line 238 ...*

Well done in spotting this; *a priori* we are reporting a ‘disagreement rate’ rather than an ‘error rate’.

Line 264-266: this assertion is not supported by previously reported results ...

We have removed this sentence.

*Lines 271-272: similarly to a statistical test where you accept a false discovery rate (e.g., risk alpha = 5%), you could compute and indicate the risk probability of declaring two accessions distinct whereas they are actually not while exhibiting ‘n’ discordant SNPs as follows: (error rate)^n. According to the error rate you consider (line 259: 0.42% - 2.9%), this probability will vary. E.g., for a conservative assessment (error rate = 2.9%), the risk probability of false discrimination between 2 accessions with 2 discordant SNPs is: (0.029)^2 = 0,000841 This computation does not account for close relatedness ... Other more elaborated statistics exist.*

Thank you very much for that, however we think your estimate of error is incorrect. When comparing two identical accessions of a variety, if the probability of incorrectly calling the haplotype of one is 0.029, then we can say that the probability of *correct* assignment is 1 - 0.029 = 97.1 %. The probability of both (genetically identical) members of this paired comparison being correctly called as the same entity is then .971 ^2 = 0.943 (94.3%).

However, ours is a practical tool rather than for use in theoretical studies. Therefore, we wouldn’t be happy if our SNP panel produced distinct SNP profiles for two accessions that are the same variety. If there was any doubt, we would repeat the assay for the given lines. We want this to be a practical tool that gives a distinct ‘fingerprint’ for every variety in a collection.

*Lines 297-298: it could also be the reverse situation, i.e. an error in SSR data or in collecting the correct material at the time of leaf collection by F. Fernandez-Fernandez ...! Here again, where is the truth? : in SSR data or in SNP data ? It can vary from case to case ...*

We appreciate that there may be errors in both our analysis as well as that of Fernandez-Fernandez. However, we were leaning on the side of caution and saying that, if other studies are correct, what is a realistic estimate of our error rate.

*Line 302: first, these results should be previously indicated in Results section and not only here; second, this worksheet ‘DEFRA comparison’ is not present in Excel table S1 ...*

Sorry for this error on our behalf. The DEFRA comparison was removed and replaced with a comparison with MUNQ codes. For the Brogdale collection, the assignment of the latter was based to a significant extent on the work carried out for the DEFRA report.

*Lines 304-309: again this paragraph should be in the Results section and not here as it is. Then, what is your interpretation (Discussion section) of these results??*

This section has been moved to the Results section.

*Lines 327-330: the preferential position of many triploids in some particular clusters is probably a matter of chance. Moreover the clustering of triploids and their position in the dendrogram rely on “incomplete”genotyping data (since the AAB and ABB genotypes are gathered in a single genotypic class AB). Their position should therefore be considered with caution.*

We agree, and that is why we point out that ‘*based upon the number of markers used, we acknowledge that any given dendrogram would not be a true representation of the relationship between the accessions studied*’. However, it is a convenient method for visualising the clusters of accessions with the same SNP profile. The purpose of carrying out the work and writing the manuscript was to demonstrate that a minimal marker set would be a relative cheap and fast, but reliable, method for use in the curation of plant collections. We don’t suggest that it should be used to study phylogeny or heterozygosity. It was interesting, however, that, even with such a small number of markers, a suggestion of the level of ploidy was apparent.

*Line 342: SNP-array-based genotyping is indeed more expensive than the simple set of 21 SNPs described here but is however much more informative. For example, pedigree relationships can be inferred from the 20K array, which will not be the case with only 21 SNP.*

We agree entirely. As mentioned above, if one were renewing an orchard and wanted to be sure that accessions being planted were as stated, our minimal marker approach would be sufficient (if you were to give us the leaves from the c. 2,000 accessions held at Brogdale we would, with high levels of confidence, be able to tell you from which trees they collected) but if one were carrying out a population study or, as you suggest, a study of pedigree, an alternative approach would be chosen.

*Lines 345-347: the indicated costs most probably only contain the technical costs and not the salary costs. It should be stated, with some indication of the time necessary if possible.*

It would be rather difficult to provide a concise response to this question, and this is why it is not included in the manuscript. However, we provide here an indication of what you ask for:

- Extracting the DNA takes about half a day for 2x96 plates and once that is done there is sufficient DNA for all markers and plenty to spare.
- Setting things up on the system takes time, but when it’s all in place, it does not have to be repeated, so I don’t think it would be fair to include that sort of thing
- Creating a DNA plate takes about 1 hour and requires 1 hour to dry.  Plates can be created simultaneously though so you could easily do 10 in the same amount of time. Then 1 hour to wash the tips and equipment (total 3 hours?)
- Running the woodpecker can be done in batches.  For new sample types/ new markers I will generally run in very small batches to check everything is going right, but it is more than possible to create up to 10 x 1536 plates in an hour ( e.g. 768 DNA samples against the 20 marker set).
- PCR reaction can take around 2 hours with another hour for scanning and re-cycling.
- Data processing – How long is a piece of string?  192 samples with one marker takes around 2-3 minutes to make a call. So that would be maybe an hour for all 20. Then additional time for more samples.
- Once one has a SNP profile this can be compared with the profiles in out MySQL database and have a responses within seconds. We are in the process of developing a website that will permit SNP profiles to be uploaded at checked on the fly.

*Line 360: correct “Accessions Studied” (not ‘Lines’)*

Correction made.

*Lines 361-362: if different trees with the same variety name were collected “from different orchards or gardens”, they should be called different accessions ...*

They are different accessions, but the same named variety. The accessions from Brogdale do have distinct accessions details, those from many of the smaller orchards do not so we can only use the names given to them when the samples were given to us. This is precisely the issue for which would suggest using our minimal marker panel and the whole point of the manuscript. Many people might believe they own a Bramley apple, for example, but if the SNP profile of these trees is different to that of a verified standard held in a well curated (phenotyped and genotyped) collection they don’t own a Bramley.

Lines 376-377: some minimal reference to the paper of Bianco et al. (2016) from where the polymorphic and robust SNP markers were initially extracted for the work of Harper et al. (2019) could be included here.

The paper is cited in the manuscript.

*Line 387: ’each of the 21 markers’ is not previously detailed.*

This is now mentioned in the ‘Identification of the SNP marker set’ section.

*Lines 387-390: as stated before, the accurate correspondence between the 21 selected SNP markers and the SNP_ID initially established in Bianco et al. (2016) should be clearly indicated beside the new names used here. E.g., BA06 (SNP_id = FB_AFFY_9751091 = AX-115521188).*

A column containing th

*Line 389: in S1, only ‘KASP probes’ is indicated for the worksheet: harmonize.*

Have corrected to ‘KASP probes’.

*Comments on Supplementary File 1 (Excel table S1): In S1, accession numbers seem not to coincide with the NFC coding system, i.e. year-number: e.g. 2000-008 or 1966-148 for Cox's Orange Pippin (LA): http://www.nationalfruitcollection.org.uk/full2.php?id=1330&&fruit=apple). Exact accession numbers (Accessions ID) should be added as an extra-column in S1 worksheets ‘Accessions studied’ and ‘Accessions with > 2 SNPs failed’ (and ‘Ploidy and Heterozygosity’) for allowing other research teams to further elaborate on the published SNP dataset.*

We have added the Accession numbers to the Supplementary File.

*Lines 14 and 15 of this worksheet seem inverted between Original names and Notes (M. Ordidge): i.e., see Adams Pearmain / Adamsapfel Ost. Same for lines 33-36 (Alkmene Alliston Pippin): switches with Notes (M. Ordidge)Probably useful to check the whole worksheet ...*

Thank you for picking this up. We’ve re-checked the whole document and it all seems okay now.

*Worksheets ‘Accession studied’ and ‘Ploidy and Heterozygosity’ seem to contain the same genotypic information for the 21 SNPs analyzed: useful to duplicate the information?*

I have eliminated some of the unnecessary columns but have left some of the replication.

*If the authors maintain the genotypic data only in the worksheet ‘Ploidy and Heterozygosity’, the accession code (Accession ID) should be duplicated in that worksheet to avoid misinterpretation with the column ‘Name’ (slight spelling differences, etc).*

*Supporting information Line 549: the furnished S1 Fig does not give any dendrogram but the copy-paste of an Excel file (close to the content of worksheet ‘Accessions studied’) ... ? Error when uploading this file?*

Supplementary files and figures have been amended and checked.
