# Supplementary material for: Development of a minimal KASP marker panel for distinguishing genotypes in apple collections
Source: PLoS One. 2020 Nov 30;15(11):e0242940. doi: 10.1371/journal.pone.0242940 (PMC7703965; doi:10.1371/journal.pone.0242940)
Supplement: Attachment — Submitted filename: response_to_reviewers.docx [file pone.0242940.s006.docx]

Reviewer 1

Reviewer #1: Congratulations on the great proofreading job. I have understood the approach very well. I still have some points that are not very clear and some little corrections

Thank you very much for all the clarifications, I am still not very clear about the identification issue, please, if you can clarify it for me a little more and on a personal level I appreciate it: if I provide you with 100 samples of which I do not know their origin, not your name or anything to identify them to me, do you think that SSRs, SNPs or both are more reliable?

I cannot really speak for the results with SSRs, although I assume one would get 100 distinct genotypes with SSRs if all the samples were distinct. I would also expect to get 100 distinct genotypes with our SNP panel.

To consider two synonymous samples, would it be necessary for the 21 SNPs to coincide or would an error be allowed? Or is it that in the case of a difference in a single SNP the analysis would be repeated to make sure? Thanks.

I would expect leaf samples taken from two different trees of the same variety to have the same SNP profile (synonyms are just different names of the same variety so the same applies). Obviously, if the test was completely blind (samples unmarked) and only one test was performed and an error had occurred it wouldn’t be obvious. This latter applies equally to SNPs and SSRs. So we are debating whether the intrinsic error rate for SNPs is better, worse or the same as that for SSRs. Unfortunately, I can’t answer that without doing a direct comparison. I can say, however, that our study produced remarkably similar results to those of studies based on SSRs.

A point that I do not understand well, if you can clarify it for me. In the File_S1 sheet "Accessions with Nominal Reps", I see that there are samples with the same MUNQ code, but different for SNPs, those samples were the same for SSRs and are they different for SNPs? (for example Afal and Cwmafan 1 and 2). This is considered an experimental error and would it be repeated? This is where the question arises again, if we did not know that they had the same name, would the analysis also be repeated? is the point that I can't quite understand.

Unfortunately, because we are not making a direct comparison with SSRs, it’s hard to answer this question with a great degree of faith. Given that we handled almost 2,700 samples it is not impossible that we made a handling error at some point, just as it is not impossible (in fact we see this in the earlier SSR-based studies of the Brogdale collection) that accessions in orchards have been mislabelled. So, yes I would want to re-sample the two ‘*Afal y* *Cwmafan*’ trees and repeat the genotyping before I came to any definitive conclusion about them. Remember, this manuscript is describing the technique and showing that it is valuable in such case; it is not a definitive study of the samples.


I think that from the first moment it should be made clear how many samples have been eliminated for the analysis, and from there always talk about the samples included in the analysis, because otherwise in some places it is confusing

This statement is made in the section entitled ‘Genotyping accuracy’; lines 213 to 223.

LINE 41: “To test the efficiency of these markers we have used them to characterise circa 2,200 distinct apple varieties: 2,104 accessions from the National Fruit Collection (NFC) at Brogdale, Kent, and 143 from the Welsh Botanical Gardens at Carmarthen”: The accessions are still not very clear here. Accessions and varieties are mixed. 2200 varietes, 2104 accessions, 143 accessions? Please, see if you can correct so that all are accessions or varieties, and that the sum matches

Having read your comments and read through the manuscript again, we realise that the numbers of samples that fall into the various categories is very difficult to follow precisely (they are after all a moving targets). In part, the 193 samples that we removed because of marker failure (almost certainly related to the quality of the DNA given that the quality of the leaf material suppled to us was very variable) create problems. However, the term ‘replicate’ really muddies the water:

- Samples: the number of different extractions made.
- Accession: the number of individual trees sampled.
- Replicates: samples extracted independently (in different wells of the plates in which extractions were made) that were known, or believed to be (‘nominal replicates’), from the same variety; this category contains both technical replicates (Yeovil Sour) and biological replicates (different tree same name).

Nominal replicates, as the name implies, may or may not be replicates. The MUNQ codes (based on SSR data) showed that some of the ‘nominal replicates’ from Brogdale were not genuine replicates at all. Our genotyping picked these up. Obviously, for the samples taken from smaller orchards and gardens MUNQ codes were not available so, *a priori*, name was all we had to identify the sample. Our genotyping, even assuming a large error rate, clearly indicated that some of these ‘nominal replicates’ were not replicates. Thus a ‘nominal replicate’ may or may not be a ‘genuine replicate.

As for our error calling, where we had no additional data (no MUNQ coes) small differences between 2 ‘nominal replicates’ could not be apportioned to an error in SNP calling or an error in naming the sample. Our worst-case error calling estimates had to carry this error.

We have tried to reword a little to avoid some of these problems.

LINE 58: 2675 TREES; (Brogdale 2,104+ Llanarthney 143+VARIOUS 429 =2676). I still do not understand the number of samples; in Table S1 2482 accessions appear collected. What do the 193 missing accessions correspond to?

As stated in the ‘Genotyping accuracy’ section of the results, only the 2,482 samples are reported in the Supplementary files.

The 2,058 ‘singletons’ are reported in Supplementary File 1.

LINE 64: “For 2,058 of these, a single tree was sampled. For 229 varieties, samples were taken from more than one listed under the variety name (often these were from different orchards or gardens)”. Are these 2058 indicated in any excel sheet?. 229 are the repetitions collected in the sheet "Accessions with Nominal Reps" (because (2rep* 125) + (3 rep * 47) + (4 rep* 16) + (5 rep*12) + (7 rep *1) + (8 rep *1) + (10 rep *1) + (23 rep *1) is not 229 or am I misunderstanding references to samples and repeats

You are mixing up two different points of the manuscript. We begin by talking about 2,058 samples that, based on the name supplied with the sample, would appear to be singletons. The numbers in File S1 spreadsheet ‘*Accessions with Nominal Reps*’ is based on the genotyping of the samples (that is the number of distinct SNP profiles). Here, of course, samples that were thought to be singletons proved to have replicates, and samples that were thought to have replicates proved to be unique. This is what is important to note: names provide a very poor method of cataloguing what is held in a collection. Our SNP panel gives a better picture (as would SSR markers).

LINE 66: “From the cultivar Yeovil Sour, 24 replicates were taken from a single tree to test reproducibility”. In the table 23 appears, which is correct? I understand that these numbers are after eliminating the samples that gave error, if so, it is interesting to clarify it here because otherwise it leads to confusion

We have clearly pointed out that one of the Yeovil Sour samples was removed from the study before the major analysis began.

Reviewer 2

2d review of the Manuscript N°: PONE-D-20-23044_R1 New manuscript Title: Development of a minimal KASP marker panel for distinguishing genotypes in apple collections Authors: Winfield M., Burridge A., Ordidge M., Harper H., Wilkinson P., Thorogood D., Copas L., Edwards K.J., Barker G.L.A. General comment: the comments previously sent in the 1st review have been considered, integrated or commented. Minor, but important points still to be considered are:

Line 117 : « here had been genotype previously” : correct ‘genotyped’

Corrected

Replicates: looking at the comments of Reviewer#1, I suspect there is an issue with the term ‘replicate’ which is used in different ways according to the situations and sentences. I propose that the authors use ‘technical replicates’ for sample replicates from a same tree, such as for the cultivar Yeovil Sour (Lines 66-67), and ‘biological replicates’ when samples come from different trees (mostly with the same names). Then, the authors should clearly define in Mat&Meth what they call ‘nominal replicates’, which adjective suddenly appears in lines 240 and 310 for sheets of Supplementary File S1 and in the caption of Table3 without having being previously defined ... This would help clarifying the terms used along the manuscript.

Lines 122-123, I would propose to change accordingly: “True replicate samples were expected to have 21 identical SNP calls; any SNP difference between technical or biological replicates was assumed, therefore, to be the result of an error in calling ...”

Changed as suggested

Line 131: “same name could be taken as putative biological replicates ...”

Added ‘putative’

Line 134: “that had fewer than 3 SNP differences could be taken as genuine biological replicates.”

Added ‘biological’

Lines 148-149: “the two samples of Kim are not true biological replicates ...”

Added ‘true biological’

Line 239: “ ... singletons and 204 with nominal replicates ...”

I don’t feel this should be changed; the sentence begins with ‘The names of the 2,482 accessions … indicate’ so it is clear that the two categories are based purely on names and NOT on genotypes. The following sentence then shows how wrong that classification is.

Lines 244-246: “the number of samples with biological replicates ... when samples have known nominal replicates, e.g. the sample Black Vallis 1 »

Added ‘biological’

Line 304: “The samples with true technical or nominal replicates allowed us ..” (since the 1stexample is for Yeovil Sour where technical replicates have been sampled)

This sentenced has been modified to indicate that we have technical reps of Yeovil Sour rather than biological reps.

Lines 308-336: again it is biological or nominal replicates and not technical ones ...

Added ‘putative biological’

Line 311: “the potential nominal replicates were collected from different trees, often from different orchards or gardens. » : it fits exactly with the definition of a biological replicates : different samples from different tress, and not from a same tree.

We are talking about putative biological replicates here (they are putative at this point in the study because we are basing the statement on names not genotypes/SNP profiles)

Lines 312-314: “The ten nominal replicates of Cox’s Orange Pippin were identical to each other, as were the eight nominal replicates of Breakwell’s Seedling, and the seven of Frederick. However, some nominal replicate samples did not have identical SNP profiles ...”

Added ‘nominal’

Lines 317-318: “In 11 cases (8.8%), potential biological (nominal) replicates had one SNP difference and in two cases (1.6%) the potential biological replicates had two SNP differences ...” Including the adjective ‘potential’ (or ‘deemed’?) has the advantage to introduce the idea that the samples are expected to be biological (nominal) replicates, but it’s not always the case ... :

Added ‘nominal’ (We were sort of hoping that, by this point in the manuscript, readers would have caught onto the idea that names are a poor guide to things being the same.

Line 324: “nine of the potential biological replicates (i.e., by name = ‘nominal’) from the NFC were confirmed not to be so” (instead of the term ‘apparent’ ...)

Removed ‘apparent’ and replaced with ‘nominal’.

Line 344: why using ‘named replicates’ here instead of ‘nominal replicates’ ??

Changed to ‘nominal’

Line 355: “In total, we had 563 accessions with true nominal replicates by name, ...” And so on ... Line 105-106: “primer sequences, together with their corresponding SNP Affymetrix code from Bianco et al. (2016) are available in File S1: spreadsheet ‘KASPprobes’. It’s very important to clearly state and highlight here the correspondence between the new KASP probes and the previous Affymetrix SNP codes

Lines 137-138: since additional MUNQ codes have been provided through the new cited reference n° 12, this reference should be used here instead of ‘personal communication’: “additional MUNQ codes, created as part of an extension of the worked initiated in (11, 16), were provided by Caroline Denancé, INRAE, Angers, France (12).” Similarly Line 325: “... been genotyped previously (20, 21) and had been assigned unique codes, MUNQ codes (11, 12) ...”

Removed ‘personal communication’ and added citation

Lines 148-149: “the two samples of Kim are not true biological replicates and we only have two true nominal samples of Gavin”

Changed to ‘are not biological replicates’

Lines 184-185: “The sequences of these 21 KASP probes, along with the corresponding SNP Affymetrix code (AX......) from Bianco et al. (2016) and the chromosomes to which they are assigned, are given in File S1” (same argument as before to allow the reader to trace the correspondence from the paper of Bianco et al. (2016) through the paper of Harper et al. to the current one ...)

Line 409: “according to analysis with flow cytometry and/or SRS markers (20)” : I guess the authors mean SSR.

Changed to ‘SSR’

Lines 423-431: simplify caption of Fig. 5 : - The following sentence “In most cases, triploid accessions correspond with high heterozygosity; there is a tendency for these samples to be found in clusters 4 (light blue) and 7 (red)” should be discarded and included in the Result section: a figure caption does not have to contain any result interpretation ... ! - Same issue for the two following sentences: “B) There was a statistically significant difference between the mean heterozygosity of triploids and that of the other three categories as determined by one-way ANOVA (F(3,2478) = 206.3, p = 2.2e-16). The unknown samples ranged in heterozygosity from very low to very high.” All that text corresponds to result interpretation, not to a fig. caption describing the figures ... !!

Fig. 5 legend has been simplified.

Lines 446-455: discussion about the possible null allele issue should be at least evoked here for BA07b, BA22 (and BA08) ! The term ‘null allele’ is yet never mentioned in the text whereas it’s a commonly used term for SNP issue in apple genetics.

Added a sentence to indicate that this might be the result of a null allele.

Lines 466-468: “Also, additional polymorphisms surrounding the target SNP may reduce probe hybridization efficiency resulting in decreased signal intensity from the fluorochrome, and possibly to null allele (11,25).”

We have extended the sentence to include ‘possibly to a null allele’.

Lines 471-474 (“shared missing marker BA08 in the accessions with MUNQ code 901 and the shared missing marker BA06 in the accessions with MUNQ code 803”) looks to be a summarized description of newly-added paragraph in Lines 481-486 : please, simplify.

The sentence has been removed.

Line 529: “... were able to detect a difference in heterozygosity between diploids and triploids...” (and not tetraploids, I guess

Yes, I think we might expect to see less heterozygosity in a autotetraploid then in an allotriploid
